# Meningococcal Sepsis in Patient with Paroxysmal Nocturnal Hemoglobinuria during Pegcetacoplan Therapy

**DOI:** 10.3201/eid3103.241182

**Published:** 2025-03

**Authors:** Leo Starck, Vuokko Nummi, Eira Poikonen, Anna-Elina Lehtinen, Pia Kivelä, Nathalie Friberg, Jukka Torvikoski, Maija Toropainen, Seppo Meri

**Affiliations:** Author affiliations: University of Helsinki, Helsinki, Finland (L. Starck, S. Meri); Helsinki University Central Hospital, Helsinki (V. Nummi, E. Poikonen, A.-E. Lehtinen, P. Kivelä, N. Friberg, J. Torvikoski, S. Meri); Finnish Institute for Health and Welfare, Helsinki (M. Toropainen)

**Keywords:** Meningococcal, sepsis, paroxysmal nocturnal hemoglobinuria, pegcetacoplan, *Neisseria meningitidis*, complement, bacteria

## Abstract

Complement C5 inhibitors bring an increased risk for *Neisseria* infections. A novel complement C3 inhibitor, pegcetacoplan, was recently approved to treat paroxysmal nocturnal hemoglobinuria, a condition commonly treated with complement C5 inhibitors. We present a case of meningococcal sepsis in a pegcetacoplan-treated patient with aplastic anemia and paroxysmal nocturnal hemoglobinuria.

Paroxysmal nocturnal hemoglobinuria (PNH) is a rare, acquired disorder caused by a de novo mutation in the *PIG-A* gene. This mutation occurs in a hematopoietic stem cell and causes a clonal loss of the glycosylphosphatidylinositol anchor from the surfaces of affected blood cells (*1*). Complement decay-accelerating factor (*2*) and protectin (*3*) are bound to cell membranes by the glycosylphosphatidylinositol anchor. Thus, glycosylphosphatidylinositol-deficient blood cells are vulnerable to complement attack, leading to hemolytic anemia, thrombi, and other PNH-related complications.

Complement inhibition is a logical therapy for PNH. In 2007, the US Food and Drug Administration and the European Medicines Agency licensed an anti-C5 monoclonal antibody, eculizumab, and in 2018–2019, the longer-acting ravulizumab. Deficiencies of the terminal complement pathway increase the risk for *Neisseria* infections (*4*). C5 inhibitor decreases the ability of human blood to kill serogroup B meningococci despite vaccination (*5*); investigators have described sepsis caused by nonencapsulated *N. meningitidis* in a patient with PNH during ravulizumab therapy (*6*).

In 2021, pegcetacoplan was the first C3 inhibitor authorized by the US Food and Drug Administration and the European Medicines Agency. Phase III clinical trial data and postmarketing safety surveillance studies revealed no invasive *N. meningitidis* infections in patients (*7*–*9*). We describe invasive *N. meningitidis* infection in a patient with PNH during pegcetacoplan therapy.

## Case Report

A 16-year-old girl received a diagnosis of aplastic anemia and PNH. The next year, her granulocyte PNH clone size increased from 1% to 98%. To address chronic, severe hemolysis, physicians prescribed ravulizumab therapy in April 2023. The patient received a dose of meningococcal conjugate vaccine (covering serogroups A, C, Y, and W-135 and a dose of meningococcal group B vaccine (4CMenB) in April 2023; she received a dose of each again in June 2023, ≈8 months before meningococcal sepsis onset. In December 2023, physicians switched the patient’s therapy from ravulizumab to pegcetacoplan (1,080 mg 2×/wk) because of extravascular hemolysis; the last ravulizumab infusion was 78 days before the onset of her infection, corresponding to 1.5 times the half-life of ravulizumab. The patient’s medications for aplastic anemia included eltrombopag (125 mg 1×/d), cyclosporine (100 mg 2×/d), and folic acid (5 mg 1×/d). She also used lynestrenol. At a follow-up appointment in January 2024, the patient’s test results showed leukopenia: total leukocyte count of 1.8 × 10^9^ cells/L (reference range 3.4–8.2 × 10^9^ cells/L) and neutrophils at 0.60 × 10^9^ cells/L (reference range 1.5–6.7 × 10^9^ cells/L).

In February 2024, three days after a student celebration on an international cruise ship, the patient, then 18 years of age, visited the emergency department with signs of septic infection: occipital headache, fever 39.5°C, tachycardia 110 beat/min, and blood pressure 105/67 mm Hg. Physical examination revealed no petechiae; thorax radiograph and urinalysis results were unremarkable. We performed blood culture and started the patient on antimicrobial therapy (cefuroxime 1.5 g 3×/d). Tests showed a total leukocyte count of 2.5 × 10^9^ cells/L and neutrophils at 1.90 × 10^9^ cells/L. The patient’s hemoglobin level was 86 g/L (reference 117–155 g/L), and lactate dehydrogenase (LDH) was 398 U/L (reference 115–235 U/L) (Tables 1, 2).

**Table 1:** Blood results and body temperature of case patient from study of meningococcal sepsis in patient with paroxysmal nocturnal hemoglobinuria during pegcetacoplan therapy*

Parameter	Reference range	Day −29	Day 0	Day 1	Day 2	Day 3	Day 4	Day 5
Hemoglobin, g/L	117–155	94	86	73†	72†	80	76†	99
Total leukocytes, × 10^9^ cells/L	3.4–8.2	1.8	2.5	1.8	3.1	2.3	2.2	3.4
Neutrophils, × 10^9^ cells/L	1.5–6.7	0.60	1.90	1.46	2.02	1.33	0.89	1.81
CRP, g/L	<4	<4	21	67	202	102	54	32
LDH, U/L	115–235	362	398	463	726	619	493	452
Fever, °C	36–37	NA	39.5	40.1	<38	<37.5	NA	NA

*Day 0 indicates day patient sought hospital care. CRP, C-reactive protein; LDH, lactate dehydrogenase; NA, not available. †Packed red blood cell transfusions.

**Table 2:** Case patient diagnostic and hospital course highlights from study of meningococcal sepsis in patient with PNH during pegcetacoplan therapy*

Treatment day	Microbiology	Treatment	Diagnostic tests and additional treatments
Day 0	Blood culture	Cefuroxime 1.5 g × 3	
Day 1	Positive blood culture; Gram staining, challenging interpretation; BCID2 PCR, negative	Cefuroxime 1.5 g × 4	Pegcetacoplan 1,080 mg; prophylactic enoxaparin started; PRBC transfusion
Day 2	Identification (MALDI-TOF mass spectrometry),* Neisseria meningitidis*; in-house PCR, negative; serogrouping, inconclusive results; preliminary report, nongroupable *N. meningitidis*	Ceftriaxone 2 g × 2	PRBC transfusion, contact tracing, prophylactic antimicrobial drugs distributed
Day 4	NA	NA	Brain magnetic resonance imaging (PNH), PRBC transfusion
Day 5	NA	NA	Pegcetacoplan 1,080 mg
Day 9	Whole-genome sequencing, nonencapsulated *N. meningitidis* harboring capsule null locus	NA	NA

*Day 0 indicates day patient sought hospital care. BCID2, bioMérieux (https://www.biomerieux.com) Biofire Blood Culture Identification 2 Panel; MALDI-TOF, matrix-assisted laser desorption/ionization time-of-flight; NA, not applicable; PNH, paroxysmal nocturnal hemoglobinuria; PRBC, 1 unit packed red blood cells.

During the first inpatient morning, the patient’s temperature peaked at 40.1°C, but she remained hemodynamically stable and in good consciousness. Her LDH increased to 463 U/L and hemoglobin decreased to 73 g/L. A transfusion of 1 unit of packed red blood cells was administered. Pegcetacoplan was continued at the same dose. Thromboprophylaxis with enoxaparin was initiated. Approximately 24 hours after admission, the patient’s blood culture sample became positive, with an unclear finding in gram-staining. A commercial multiplex-PCR array (Biofire FilmArray Blood Culture Identification Panel 2; bioMérieux, https://www.biomerieux.com) yielded no identification. Dosing of cefuroxime was increased to 4 times a day.

On the second day, matrix-assisted laser desorption/ionization time-of-flight mass spectrometry (VITEK MS; bioMérieux) identified *N. meningitidis,* but capsule agglutination was inconclusive. An in-house meningococcus-specific PCR remained negative. The multiplex PCR repeatedly returned a negative result for all targets. Cefuroxime was switched to ceftriaxone (2 g 2×/d), a medication more appropriate for treating meningococcal sepsis. Subsequent laboratory tests revealed a decrease in hemoglobin to 72 g/L and an increase in LDH to 726 U/L, indicating active hemolysis. She received another infusion of packed red blood cells. 

Epidemiologic contact tracing and distribution of antimicrobial prophylaxis to close contacts commenced. The meningococcal isolate was sent to the Finnish Institute for Health and Welfare for verification, serogrouping, and characterization by whole-genome sequencing that included genes of the 4CMenB vaccine antigens. The isolate auto-agglutinated and was not groupable using a latex agglutination test (BioRad Laboratories, https://www.bio-rad.com).

On the third day of her hospital stay, the patient remained clinically stable, and her LDH level decreased to 619 U/L. On the fourth day, a slight drooping of the left side of her mouth was noticed. Magnetic resonance imaging of her brain revealed no abnormalities. The patient recovered fully and was discharged in good health after a 7-day course of intravenous antimicrobial drugs. She continued pegcetacoplan therapy for PNH. At discharge, the patient was prescribed amoxicillin/clavulanate (875/125 mg) to be started in duplicate in case of a sudden onset of fever at distance from hospital. On a follow-up visit for PNH, the patient gave informed consent for publication of a case report.

Whole-genome sequencing revealed a nonencapsulated *N. meningitidis*, harboring the capsule null locus (cnl) allele 2. The strain designation of the isolate was sequence type 198, clonal complex 198 (cnl: P1.18,25–1: F5–5: ST198 (cc198) (capsule group:PorA type:FetA type:sequence type [clonal complex]). The isolate contained neisserial heparin binding antigen peptide 10 and factor H binding protein peptide 94 but lacked neisserial adhesin A. The genetic Meningococcal Antigen Typing System (*10*) predicts isolate coverage by 4CMenB because of cross-reactive neisserial heparin binding antigen. Blocking of complement C3 probably hinders killing by both opsonophagocytosis and the complement terminal pathway (*11*), although there is also evidence for serum killing during C3 blockade through a C3 bypass pathway (*12*). The negative diagnostic PCR and latex agglutination test results were likely due to lack of capsular transport and biosynthesis genes, including the PCR-targeted *ctrA* (*13*).

Infections caused by nonencapsulated *N. meningitidis* are rare. The epidemiology of cnl invasive meningococci is difficult to ascertain. The 2022 European Centre for Disease Prevention and Control Surveillance Report for Invasive Meningococcal Disease (*14*) listed serotypes A, X, Z, 29E, nongroupable, and others in the same category (n = 63 in Europe in 2022). The US Centers for Disease Control and Prevention categorized cnl meningococci as nongroupables (n = 30) and other/unknowns (n = 40) in 2022. The PubMLST database (https://pubmlst.org/neisseria), however, received >100 reports of invasive infections by cnl meningococci, most belonging to the same strain designation as the isolate infecting our patient. As whole-genome sequencing becomes more common, allowing a more detailed characterization than the agglutination test, cnl and other nongroupable meningococci will likely become more frequently recognized and differentiated.

## Conclusions

Our patient had aplastic anemia and associated PNH, with relative leukopenia and complement inhibition, increasing her susceptibility to meningococcal infections. Iatrogenic complement inhibition with pegcetacoplan and potential residual ravulizumab predisposed this patient to an unusual septic infection by nonencapsulated *N. meningitidis*, despite immunization with 4CMenB 10 months and 8 months earlier. We faced some clinical challenges in diagnosing and treating this patient. First, several meningococcal identification tests returned negative results. Second, because complement C3 inhibitor therapy hampers protection against meningococci conferred by 4CMenB immunization, patients who suffer severe infections must sometimes compromise continuity of complement inhibitor treatment. Finally, with evidence lacking about efficacy and safety of antimicrobial prophylaxis in complement inhibitor recipients (*15*), physicians lack guidance regarding appropriate prophylactic antimicrobial drugs after severe infections in patients with PNH. Our case sheds light on the obstacles involved in both diagnosing and treating neisserial infections in complement inhibitor recipients and demonstrates the importance of vigilance and rapid administration of effective antimicrobial drugs.
